# Neonatal survival in complex humanitarian emergencies: setting an evidence-based research agenda

**DOI:** 10.1186/1752-1505-8-8

**Published:** 2014-05-20

**Authors:** Diane F Morof, Kate Kerber, Barbara Tomczyk, Joy Lawn, Curtis Blanton, Samira Sami, Ribka Amsalu

**Affiliations:** 1 Division of Reproductive Health, Centers for Disease Control and Prevention, 4770 Buford Hwy, MS-F-74, Atlanta, GA 30341, USA; 2 Saving Newborn Lives, Save the Children, 28 Lower Main Road, Observatory 7925, South Africa; 3 University of the Western Cape, Bellville 7535, South Africa; 4 Emergency Response and Recovery Branch, Centers for Disease Control and Prevention, 4770 Buford Hwy, MS-F-57, Atlanta 30341GA, USA; 5 Epidemiology, London School of Hygiene and Tropical Medicine, Global Evidence and Policy, Saving Newborn Lives, Save the Children, Keppel Street, London WCIE-7HT, UK; 6 Save the Children, 54 Wilton Road, Westport, CT 06880, USA

**Keywords:** Neonatal, Newborn, Research, Priority-setting, Neonatal infections, Preterm birth, Birth asphyxia, Inequity, Epidemiology, Millennium development goals, Complex humanitarian emergency

## Abstract

**Background:**

Over 40% of all deaths among children under 5 are neonatal deaths (0–28 days), and this proportion is increasing. In 2012, 2.9 million newborns died, with 99% occurring in low- and middle-income countries. Many of the countries with the highest neonatal mortality rates globally are currently or have recently been affected by complex humanitarian emergencies. Despite the global burden of neonatal morbidity and mortality and risks inherent in complex emergency situations, research investments are not commensurate to burden and little is known about the epidemiology or best practices for neonatal survival in these settings.

**Methods:**

We used the Child Health and Nutrition Research Initiative (CHNRI) methodology to prioritize research questions on neonatal health in complex humanitarian emergencies. Experts evaluated 35 questions using four criteria (answerability, feasibility, relevance, equity) with three subcomponents per criterion. Using SAS 9.2, a research prioritization score (RPS) and average expert agreement score (AEA) were calculated for each question.

**Results:**

Twenty-eight experts evaluated all 35 questions. RPS ranged from 0.846 to 0.679 and the AEA ranged from 0.667 to 0.411. The top ten research priorities covered a range of issues but generally fell into two categories– epidemiologic and programmatic components of neonatal health. The highest ranked question in this survey was “What strategies are effective in increasing demand for, and use of skilled attendance?”

**Conclusions:**

In this study, a diverse group of experts used the CHRNI methodology to systematically identify and determine research priorities for neonatal health and survival in complex humanitarian emergencies. The priorities included the need to better understand the magnitude of the disease burden and interventions to improve neonatal health in complex humanitarian emergencies. The findings from this study will provide guidance to researchers and program implementers in neonatal and complex humanitarian fields to engage on the research priorities needed to save lives most at risk.

## Background

The push to achieve Millennium Development Goal (MDG) 4 —the reduction by two-thirds of the under-five mortality rate (U5MR) by 2015—has drawn greater attention to child survival. Over 44% of all deaths among children under five are neonatal deaths (0–28 days), and this proportion is increasing as deaths decline more rapidly amongst children after the first month of life [1,2]. In 2013, nearly three million newborns died, with almost all (99%) occurring in low- and middle-income countries [2]. Approximately three-quarters of these deaths take place in the first week of life with more than a third dying within 24 hours of birth [3-5]. The majority of these deaths are due to preventable and treatable causes. Direct preterm birth complications are the leading cause of newborn deaths and the second leading cause of all under-five mortality. Preterm birth complications, intrapartum-related deaths, and infections account for more than 80% of deaths globally [6]. If mothers and newborns receive quality care over two-thirds of newborn deaths could be averted worldwide [7]. Newborn survival and health in resource-poor settings has recently emerged on the global health agenda with a resulting push to define the problem and generate evidence on best practices for care [8] but this agenda has not translated to emergency settings.

While under-five mortality rate (U5MR) has been decreasing globally [9-11], progress is slowest in sub-Saharan African countries. Many of the countries with the highest neonatal mortality rates are currently or have recently been affected by complex humanitarian emergencies (see Table 1) [12][13-15]. Complex humanitarian emergencies are due to conflict, war or civil disturbance, natural disasters, food insecurity or other crises that affect large civilian populations and result in significant excess mortality. These crises are often characterized by population displacement as well as the collapse of basic health services and local and national infrastructure that results in the need for international assistance as the emergency exceeds the capacity of the local government [16].

**Table 1:** **Conflict and refugee status in the 25 countries with the highest neonatal mortality rate (NMR)**[2,13,14,17]

**Rank by highest neonatal mortality rate**	**Country**	**Neonatal mortality rate/1,000 live births (UNICEF 2013)**	UCDP (2011 and 2012) any conflict*	**UNESCO (2010) report definition of conflict**^***ǂ***^	**UNHCR (mid-2013) Number refugees from this country**	**UNHCR (mid-2013) Number of refugees in this country**	**UNHCR (mid-2013) IDPs in this country**
			**Includes any: war and minor conflict, non-state conflict and one-sided violence**				
1	Mali	59	Yes		182,780	14,425	353,455
2	Sierra Leone	59	No	Post conflict	5734	4154	0
3	Guinea-Bissau	58	No		1,162	7,787	0
4	South Sudan°	57	Yes	N/A	102,651	223,636	401,433
5	Pakistan	56	Yes	Armed Conflict	46,046	1,621,525	975,478
6	Ethiopia	54	Yes	Armed Conflict	73,926	406,646	0
7	Guinea	54	Yes	Post conflict	13,956	10,466	0
8	Mozambique	54	No		63	4,413	0
9	Bangladesh	54	No		9,545	231,138	0
10	Nepal	53	No	Armed Conflict	7,323	51,232	0
11	Nigeria	52	Yes	Non-State	17,735	1,849	0
12	Angola	52	No	Post conflict	16,229	23,718	0
13	India	51	Yes	Armed Conflict	11,784	187,024	0
14	Liberia	51	No	Post conflict	22,488	58,852	0
15	Malawi	50	No		275	6,369	0
16	Somalia	50	Yes	Armed Conflict	1,130,939	2,339	1,122,559
17	Afghanistan	50	Yes	Armed Conflict	2,552,208	16,866	5,367
18	Niger	48	No		657	50,424	0
19	Côte d’Ivoire	48	Yes	Post conflict	93,738	4,025	24,000
20	Democratic Republic of Congo	47	Yes	Armed Conflict	490,095	183,244	2,607,407
21	Equatorial Guinea	47	No		214	0	0
22	Timor-Leste	47	No	N/A	N/A	N/A	N/A
23	Central African Republic	47	Yes	Extra definitional	221,577	17,732	206,000
24	Chad	47	No	Armed Conflict	39,329	418,451	90,000
25	Burundi	46	No	Armed Conflict	73,143	44.034	78,948

*Includes any: war and minor conflict, non-state conflict and one-sided violence. Data from 2011 and 2012.

^*ǂ*^UNESCO report definitions based on battle-related deaths: from Armed Conflict Dataset or the Battle Death Dataset 1990–2008. Conflict-affected means two things: The problems caused by an ongoing or very recent conflict and the problems that associated with a post-conflict country. A conflict defined as active will have produced more than 1000 BRD for the period 1999–2008 in addition to more than 200 BRD in any of the years 2006, 2007 or 2008. If a conflict meets the former criterion but not the latter we classify it as a post-conflict country. Extra definitional was used to denote that conflict was known to occur in this country but did not meet the criteria for each category stated above.

°South Sudan seceded from the Republic of Sudan in 2011. IDP figure in South Sudan includes 209,700 people who are in an IDP-like situation. An unknown number of refugees from South Sudan may be included under Sudan (500,014) (in absence of separate statistics for both countries).

In 2011 it was estimated that 42.5 million people were displaced by events such as war and natural disasters [17]. Women and children often make up a majority of the displaced population and account for a large proportion of disability, morbidity and mortality among this population [18]. The main adverse health consequences within this group are due to poor pregnancy outcomes that affect both the mother and newborn, and to the increased risk of sexual violence and exposure to HIV/AIDS [16,19,20].

Each humanitarian response situation varies enormously in how efficiently and equitably health services are provided. Many areas of humanitarian response are undergoing a process of reform based on operations research and information exchange on minimum standards of care and consistent principles for providing health services. For example, as a result of increasing knowledge on best practices for neonatal survival, particularly in developing countries, this issue has recently been emphasized in research and policy statements as an essential component of humanitarian response [21-23]. While guidelines exist on what to do to improve maternal and newborn health [24,25], it is less clear on how to implement those practices systematically across settings [15].

### Neonatal health in complex humanitarian emergencies

Few published studies exist on the burden of neonatal health in complex humanitarian emergencies and even fewer provide information on improving service delivery in these settings. Therefore, a literature review was undertaken to understand the epidemiological burden of neonatal mortality in complex humanitarian settings [Adler A, Amsalu R, Kerber K, Lawn J, Neonatal Mortality Literature Review unpublished]. Using 24 specific search terms, researchers identified 34 reports from 17 different sources including published papers, gray literature, unpublished work by epidemiologists, and digital and hard copy review of documents, Demographic Health Surveys (DHS) [26], Multiple Indicator Cluster Surveys (MICS 3) [27], and the International Disaster Databases [28]. Key findings from this review showed most mortality surveys included crude morality rate (CMR) and U5MR, with very few surveys identifying neonatal mortality rates or causes specific to newborns. Additionally, DHS and MICS surveys from conflict-affected countries were reviewed to determine if data were disaggregated for the portion of the population that was internally displaced and found that few countries had that information available. The lack of epidemiologic information on the causes of neonatal disability, morbidity, and mortality and effective interventions in complex humanitarian emergencies prompted a discussion on the need for more data on neonatal health in these settings.

### Child Health and nutrition research Initiative method

Funding for public health research in complex humanitarian settings, much like many other settings, is limited [29]. Given the scarcity of resources, the Child Health and Nutrition Research Initiative (CHNRI) developed a systematic method to align health research investments with the burden of global child health and the potential impact of the research by having key stakeholders identify priority research questions [30-32]. The CHNRI method is a flexible process and includes up to 15 steps that enables prioritizing health research investments at the institutional, regional, national, international, or global level. The CHNRI methodology has been used to rank research priorities for preventing stillbirths [33] and for many other subject areas such as intrapartum-related neonatal deaths, newborn infection, preterm and low birth rate, zinc deficiency in children, as well as mental health and disability [29,34-45]. Using the CHNRI method has the additional potential to engage researchers, donors, humanitarian agencies and health professionals to improve dialogue and sustain and expand beyond the research selection process to form part of a viable public health strategy [29,37,39-41,46].

The purpose of this study was to use an adapted CHNRI methodology to prioritize neonatal health research most likely to reduce newborn related mortality in the unique setting of complex humanitarian emergencies. The purpose was also to facilitate greater openness, communication and collaboration among partners and improve the allocation of research funds for neonatal health research in complex humanitarian emergencies [40].

## Methods

Save the Children and the US Centers for Disease Control and Prevention (CDC) invited technical experts to apply the CHNRI methods to systematically list research questions and use standard methods to score them (see Appendix 1 and Additional file 1). We applied the following processes: (i) Defining the research context, questions, and criteria for priority setting; (ii) Enlisting experts to systematically score the research questions, and; (iii) Computing and writing up the results.

### Defining the research context, questions, and criteria for priority setting

#### Research context

The research context was defined by space, time, population, and burden of disease. The *space* we used was complex humanitarian emergencies, defined as acute or chronic situations of conflict, war or civil disturbance, natural disasters, food insecurity or other crises that affect large civilian populations, result in significant excess mortality, and are beyond the capacity of the local government to cope [16]. *Time* for the context of the research was defined as present day to the year 2020. The *burden of disease* was all cause mortality and disability during the neonatal period. The population of interest was internally displaced persons (IDP), refugees, and communities affected by complex humanitarian emergencies in low and middle income countries.

#### Research questions

Global experts in neonatal health working on Save the Children’s Saving Newborn Lives program compiled a list of 97 research questions, some of which were used in previous research prioritization exercises for non-emergency settings. Using a research pipeline model that promotes a global perspective and intent to scale-up in both low and high-income settings [47], the research questions were categorized into descriptive epidemiology (describing the situation and understanding the determinants), leading to development (improving existing interventions by increasing deliverability or reducing cost), delivery (health policy and implementation research and scale up), and discovery (new and basic science concepts or technologies). The 97 neonatal research questions were reviewed by the experts to remove duplication as well as ensure appropriate applicability and relevance to complex humanitarian settings. A final list of 35 questions was used for the prioritization exercise. The final questions were categorized into descriptive (n = 9), development (n = 8), and delivery (n = 18). Based on expert selection, there were no discovery questions included in the final list.

#### Criterion selection

Criteria were agreed upon for use in prioritizing the research questions. These criteria were chosen based on a standard methodology used in previous prioritization exercises developed by the Child Health and Nutrition Research Initiative (CHNRI) and used for multiple exercises in research priority setting for varied subjects [37]. Each of the 4 criterion had three subcomponents (see Additional file 2).

1. Answerability: likelihood that research would lead to new knowledge in an ethical way;

2. Feasibility: likelihood that research can be conducted cost-efficiently and leads to generalizable new knowledge;

3. Relevance: likelihood that research would address important condition and critical gap in knowledge and could be readily translated to inform policies and programs

4. Equity: likelihood that the proposed epidemiological research would have positive impact on equity and local ownership.

### Enlisting experts to systematically score the research

An inclusive approach was taken and participation was widely solicited from persons with subject matter expertise (neonatal, child health, reproductive health and complex humanitarian emergencies), including both field staff and those at head offices. Initially, an email was sent to members of the Inter-agency Working Group (IAWG) on Reproductive Health in Crises sub-working group on Research, Data and Heath Information Systems and the general IAWG list serve [48]. In addition, researchers were advised to extend the invitation to others who may have appropriate technical expertise. Lastly, information about the study was presented at the IAWG 13th annual workshop where all attendees who had the appropriate expertise were encouraged to participate and they were followed up with an email invitation [49]. This activity took place between October 14 and December 10, 2011.

Participants were asked to complete the ranking by completing either a web-based survey or spreadsheet received through e-mail from research coordinator. Within the email and web link, participants were provided with the definitions regarding the space, time and disease burden of interest. The instructions asked participants to prioritize epidemiological methods for measurement and implementation research questions to achieve a reduction in the number of newborn deaths in complex humanitarian emergencies.

For each of the 35 research questions, participants were asked to review all four criteria. Each criterion had three subcomponents (see Additional file 2). Participants were asked to select “YES” or “NO” to the best of their knowledge. The written instructions in the spreadsheet or survey stated that if they felt they understood the question well and possessed knowledge to answer it, but if they felt the answer wasn’t a clear “YES” or “NO” to enter the option “YES or NO”. In the cases where participants felt they did not have enough knowledge or information to answer a question, they were instructed to select “DON’T KNOW” rather than guess.

### Data analysis

Responses were scored: “YES” (1 point); “NO” (0 points); “YES or NO” (0.5 points); and “DON’T KNOW” (missing data). If the survey was not done via the web, the completed worksheets were returned to the research coordinator. Using SAS 9.2 (SAS Institute, Cary, NC). An overall mean research prioritization score (RPS) was calculated by taking the average of the four criteria [38]. For each research criterion, the average score was computed by taking the average of the subcomponent scores. Questions were ranked in order of the highest RPS.

The overall research priority score (RPS) was computed as the mean of the scores for the four criteria, according to the input from the experts, according to the formula:

$$\mathrm{RPS}=\frac{\mathrm{mean}\;C1+\mathrm{meanC}2+\mathrm{meanC}3+\mathrm{meanC}4}{4}$$

C designates the scores for relevant criteria.

As a measure of agreement among scorers, the Average Expert Agreement (AEA) [37,38] scores were computed for each research question. The AEA was defined as the average of the highest proportion of the 12 subcomponents among scorers for each of the 28 questions asked. This gave us an indication of the most frequent response among scorers, for example an AEA measure of 0.70 would say that on average 70% agreed on the most frequent response. The AEA was computed for each scored research question as:

$$\mathrm{AEA}=\frac{1}{12}\times \sum_{\mathrm{sc}=1}^{12}\frac{N\;\mathrm{of}\;\mathrm{scorers}\;\mathrm{who}\;\mathrm{provided}\;\mathrm{most}\;\mathrm{frequent}\;\mathrm{response}}{N\;\mathrm{of}\;\mathrm{all}\;\mathrm{scorers}}$$

We conducted a sensitivity analysis to determine if the priorities changed when information was included from participants that completed the survey and those that were incomplete. We also reviewed all questions to determine the proportion of those who answered “yes/no” or “don’t know” for all subcomponents of an individual question. Additional information on methods is available in web appendices (A-D).

The Center for Global Health at the US Centers for Disease Control and Prevention reviewed the protocol and determined that the activity was not human subject’s research and the primary intent was public health response.

### Funding

No specific funding was received for this study and the time of the experts was covered by their respective institutions.

## Results

Thirty-eight experts participated and 28 experts completed the survey. Surveys were completed from October 17 until December 8, 2011. Experts were from UNHCR, UNICEF, UNFPA, Save the Children, the US government - CDC, the European Union Humanitarian Aid and Civil Protection- (ECHO), three academic institutions and nine non-governmental international organizations.

Research prioritization scores (RPS) ranged from 0.679 to 0.846; scores closer to 1.0 were considered a higher priority (see Table 2). Average expert agreement (AEA) ranged from 0.411to 0.667. Table 2 presents the priority questions ranked by RPS and the correlating AEA for those who completed the survey. The mean score for each criterion is also presented.

**Table 2:** Survey completers’ priority ranked questions (n = 28), research priority scores, average expert agreement and mean criterion scores

**Rank**	**Question**	Research type*	**Research Priority Score (RPS)**	**Average Expert Agreement (AEA)**	**Answerability**	**Feasibility**	**Relevance**	**Equity**
1	What strategies are effective in increasing demand for, and use of skilled attendance?	DEL	0.846	0.643	0.837	0.824	0.870	0.852
2	What is the feasibility, effectiveness and cost of approaches to increase coverage of clean delivery practices in facilities and in homes?	DEL	0.841	0.616	0.820	0.774	0.863	0.908
3	What is the additional burden of neonatal mortality in different emergency situations (e.g. conflict, acute vs. protracted, natural disaster)?	DES	0.833	0.667	0.836	0.755	0.883	0.858
4	Can simplified pregnancy surveillance at community level be used to measure neonatal mortality?	DES	0.830	0.616	0.799	0.857	0.797	0.867
5	Can simplified verbal autopsy tools be adapted for use in emergency settings to capture the main causes of neonatal mortality?	DES	0.828	0.661	0.827	0.789	0.825	0.872
6	Develop and validate strategies to identify preterm babies at community level by CHWs and family members	DEV	0.826	0.610	0.823	0.768	0.859	0.855
7	Which risk factors for neonatal sepsis can be identified in emergency settings and these mothers and babies given extra support? E.g. low birth weight, short gestational age, unhygienic delivery, skin and umbilical cord care, hypothermia, poor feeding practices.	DES	0.818	0.634	0.797	0.765	0.841	0.868
8	Can pregnancy surveillance at community level contribute to increased uptake of facility-based delivery?	DEL	0.816	0.610	0.866	0.815	0.790	0.793
9	What is the feasibility, effectiveness and cost of different approaches to increase coverage of syphilis screening in pregnancy, treatment and partner treatment?	DEL	0.814	0.568	0.827	0.801	0.812	0.814
10	What is the feasibility, effectiveness and cost of different approaches to promote handwashing of caregivers?	DEL	0.812	0.610	0.862	0.835	0.743	0.807
11	What is the incidence of neonatal sepsis in emergency settings?	DES	0.803	0.619	0.788	0.764	0.802	0.861
12	What is the relative proportion of death in the neonatal period to other causes of child mortality in emergency settings?	DES	0.794	0.610	0.783	0.784	0.755	0.853
13	What is the feasibility, effectiveness and cost of approaches to increase and/or maintain tetanus toxoid coverage?	DEL	0.791	0.557	0.828	0.773	0.679	0.885
14	Can use of perinatal audit reduce the incidence of adverse outcomes related in acute intrapartum events?	DEL	0.785	0.500	0.805	0.795	0.801	0.740
15	What is the feasibility, effectiveness and cost of different approaches to promote hygienic cord and skin care?	DEV	0.783	0.548	0.808	0.782	0.782	0.762
16	Evaluate ways to provide thermal care and feeding for the very preterm baby at or close to home	DEV	0.780	0.512	0.798	0.766	0.744	0.812
17	What is the feasibility, effectiveness and cost of a scheme of routine home visits for initiation of supportive practices, detection of illness and newborn survival?	DEL	0.771	0.539	0.799	0.747	0.743	0.795
18	What is the feasibility, costs and effectiveness of setting up newborn care corners in mobile clinics, first referral units and district hospitals?	DEL	0.769	0.536	0.790	0.725	0.823	0.739
19	Can simpler clinical algorithms (recognition and management) be developed and validated for babies who require resuscitation at birth, and does this increase met need for resuscitation at birth?	DEL	0.767	0.539	0.669	0.785	0.809	0.806
20	Safety, feasibility and effectiveness and cost of managing severe neonatal infections at or close to home (e.g. requiring injectable antibiotics)	DEV	0.763	0.530	0.807	0.590	0.836	0.820
21	What is the incidence, causes and outcomes of umbilical and skin infections among newborns in emergency settings?	DES	0.762	0.521	0.789	0.703	0.701	0.855
22	What is the feasibility and effectiveness of approaches to improve aseptic practices in labour rooms, maternity, paediatric wards and nurseries?	DEL	0.753	0.533	0.747	0.754	0.779	0.731
23	What is the feasibility, effectiveness and cost of approaches to increase coverage of antibiotics for prolonged rupture of membranes?	DEL	0.752	0.494	0.779	0.716	0.738	0.776
24	What is the relative proportion of neonatal infections that are pneumonia, sepsis, meningitis and are there reliable clinical markers/combination of markers to distinguish these conditions?	DES	0.739	0.521	0.686	0.720	0.734	0.815
25	What is the feasibility and effectiveness of approaches to improve quality of care in hospitals?	DEL	0.739	0.479	0.706	0.729	0.752	0.768
26	Can introduction of Doppler increase the use of partograph to monitor labor?	DEL	0.724	0.461	0.829	0.736	0.655	0.675
27	What is the attribute of clean delivery kit distribution in decision making process for home or facility based delivery?	DEL	0.713	0.429	0.729	0.695	0.677	0.751
28	What is the additional burden of stillbirth in different emergency situations (e.g. conflict, acute vs protracted, natural disaster)?	DES	0.706	0.515	0.725	0.604	0.785	0.709
29	Can low-cost, robust, simple fetal heart monitors be developed and tested that are more user-friendly than the Pinard? Does use of such a device improve fetal heart rate monitoring and reduce intrapartum stillbirth and asphyxia-related outcomes?	DEV	0.700	0.443	0.718	0.689	0.770	0.622
30	Can UTI screening in pregnancy be reduced in cost and made more feasible so can be used in lower levels of care?	DEV	0.698	0.438	0.726	0.653	0.663	0.751
31	Can simpler, cheaper technology be developed to improve supportive care of neonates who require oxygen (such as robust pulse oximeter, oxygen condensers, low cost CPAP etc.) and does this reduce deaths, improve outcomes?	DEV	0.696	0.458	0.734	0.617	0.760	0.675
32	How can diagnostic facilities in health facilities for identification of neonatal sepsis be improved?	DEL	0.696	0.461	0.662	0.682	0.758	0.684
33	What is the feasibility, effectiveness and cost of different approaches to promote prompt care seeking for illness from an appropriate provider?	DEL	0.691	0.426	0.699	0.614	0.705	0.744
34	Identification of new interventions to prevent transmission of infections during childbirth, e.g. chlorhexidine vaginal douche, immune modulators like zinc to mothers.	DEV	0.679	0.440	0.678	0.674	0.692	0.672
35	Evaluate different methods of behavior change that overcome harmful practices and promote positive cultural and social norms	DEL	0.679	0.411	0.751	0.585	0.681	0.699

*Research type: DES = description; DEL = delivery; DEV = development.

The majority of questions had only one or two respondents that selected “don’t know” for all components of a question. There was one question where eight respondents (26%) selected “don’t know” for all subcomponents; “What is the attribute of clean delivery kit distribution in decision making process for home or facility based delivery?”

The results were similar for those who completed the survey and those that did not finish the entire survey (n = 10). The ranking, based on RPS, changed for only a few of the questions in the mean score. The overall correlation was very high. The top 5 questions remained in the 5 priority questions for both those that completed the survey and those that did not.

The top 10 research questions covered a range of issues along the research continuum. Reflecting the lack of basic data to describe the extend of the problem of neonatal morbidity and mortality in these settings, four questions dealt with measurement –namely, determination of additional burden of neonatal mortality in emergency situations, pregnancy surveillance to measure neonatal mortality, verbal autopsy to capture causes of neonatal mortality and risk factors for neonatal sepsis. The programmatic priorities included a focus on safe birth, such as increasing demand for skilled attendance, coverage of clean birth practices, and facility delivery uptake. Additionally, two of the programmatic questions focused on the specific components rather than general care–specifically, hand washing and prevention of congenital syphilis. Only one development question was ranked in the top 10–develop strategies to identify preterm babies at the community level.

## Discussion

To our knowledge, this is the first systematic ranking of research priorities for neonatal survival in emergency contexts. We focused on research priorities for neonatal mortality reduction because newborns are the most vulnerable members of any population, particularly in populations affected in emergencies. The identified priorities are also of relevance for women and many also relate to older children.

Four of the top ten priority questions were identified under the “description” category, highlighting the lack of knowledge about the burden of neonatal mortality and morbidity in emergency settings. The need for defining the scope of the public health problem prior to implementing interventions is outlined in the CDC Public Health Model, which begins by describing the problem, then identifying risk and protective factors, followed by developing and testing prevention strategies, and finally disseminating prevention strategies and assurance of widespread adoption [50]. Description of the problem was prioritized here possibly because the burden of the problem needs to be better identified before opportunities for “delivery” and “development” research questions can be put forth.

The highest ranked question was, “What strategies are effective in increasing demand for and use of skilled attendance?” highlighting the importance of the time of birth and the issue of access to providers who have an appropriate level of skill. In a recent research priority ranking on reduction of stillbirths and preterm deliveries at the community level, the highest ranked question was: “Evaluate the financial barriers to facility births at the community level (user fee exemption, emergency loans, conditional cash transfers, transportation vouchers, etc.)” [32]. In both the development and humanitarian settings, the importance of increasing demand for a safe birth with trained providers is evident, but is especially challenging in emergency contexts. Two other questions in the top ten priorities relate to care at birth, but with a focus on clean birth (number 2 on clean birth kits, and number 9 on handwashing). Prioritizing hygienic care may be the first and most feasible steps towards ensuring clean and safe births in these challenging settings with limited skilled personnel and many home births.

The overall high RPS range (0.679 to 0.846) which is closer to 1.0 suggests that the majority of questions were considered high priority. It is possible that questions were reviewed and deemed high priority in part because of the initial review of the original list of questions reduced the total number to those only applicable to this setting. This range was similar to only one study thus far (range 0.56 to 0.86) [32] whereas another study showed a broader range (0.25 to 0.90) [34,36,42-44]. Additionally, similar to other research prioritization exercises, the AEA showed a direct positive association with the RPS, indicating that the agreement among experts was greater for the top ranking questions than for the lower ranking questions. High and low RPS scores represent high levels of agreement whereas a AEA closer to the mean value represents more disagreement [32,34,36,42-44]. The one question, where eight respondents selected “don’t know” for all subcomponents, “What is the attribute of clean delivery kit distribution in decision making process for home or facility based delivery?” may have not been clear to some respondents. Review of question wording with a broader audience to ensure clarity of meaning should be conducted in the future.

The CHNRI methodology provides a systematic way to evaluate and prioritize research questions, although some limitations exist. This process, while valuable, was time consuming for participants. For each of the 35 questions, 12 subcomponents needed to be assessed. This burden may be reflected in the lack of survey completion by ten of the invited participants; however, the sensitivity analysis revealed the highest ranked priorities were consistent among those who finished the survey and those who did not. It is also possible that the respondents most interested in this topic would have completed the survey and that respondent bias may exist. This exercise was conducted in English only. In the future, multiple languages and additional perspectives from those currently operating within complex emergency settings would be valuable to include in the prioritization. Although affiliations were provided for each expert, data on location of work (field-based vs. headquarters) and educational background were not obtained. All attempts were made to engage field-based staff with a broad range of educational backgrounds and expertise; however, the success of these efforts may have been limited given the challenge of accessing field-based staff in acute emergency situations in particular. Location of the participant’s primary work environment, educational background and type of occupation should be determined in the future.

The priority research questions identified in this review are timely, given the increasing focus in global health on newborn survival and health, within the context of an integrated continuum of care from pregnancy, childbirth, the postnatal period and beyond, across all levels of service delivery. The global *Every Newborn* Action Plan has identified that in order to accelerate progress for newborn health different contexts require tailored approaches, with specific attention to preparedness for, and rapid response to, complex humanitarian emergencies [51]. Filling the knowledge gaps for these settings is an overdue need which will be tracked alongside specific milestones and indicators through the *Every Newborn* process and inter-agency collaboration.

## Conclusions

Meeting the needs of pregnant women and newborns in complex humanitarian emergencies is challenging but not impossible. This ranking of priority research questions provides a clear opportunity and need to fill knowledge gaps to meet this challenge. However, the acute nature of many emergencies requires systematic capacity building before emergencies occur to ensure proven solutions can be delivered and monitored for impact in these high-risk settings. The research priorities identified here emphasize an immediate need to focus on both descriptive epidemiology as well as operations research to improve outcomes, especially around the time of birth. This exercise has already stimulated discussion and action to address gaps in research by donors, international non-governmental organizations (NGOs), national stakeholders and researchers. Additional financial and human resources dedicated to conducting research in these settings may bridge the gap and answer critical questions. Engaging partners that have expertise in neonatal health and those with expertise in emergencies to work together is critical to reducing neonatal mortality. A wide network of partners in both the development and humanitarian sphere will be needed to address these knowledge and action gaps in complex humanitarian fields and together to improve maternal and neonatal health and survival, especially amongst the women and newborns who are most at risk.

## Appendix 1

The CHNRI methodology for setting priorities in health research investments

### STAGE 1: Defining the context and criteria for priority setting

Research priority scores for many research investment options may change based on different contexts so specifying the context a priori is a critical part of the CHNRI process.

The research context was specified by space, time, population, and burden of disease context by Save the Children and the US CDC as follows:

• The *space:* complex humanitarian emergencies, defined as acute or chronic situations of conflict, war or civil disturbance, natural disasters, food insecurity or other crises that affect large civilian populations result in significant excess mortality, and are beyond the capacity of the local government to cope;

• *Time:* from present day to the year 2020;

• *The population of interest*: internally displaced persons (IDP), refugees, and communities affected by complex humanitarian emergencies in low and middle income countries;

• The *burden of disease* was all cause mortality and disability during the neonatal period.

### STAGE 2: Choice of technical experts

The research coordinator used an inclusive approach to solicit participation from persons with subject matter expertise (neonatal, child health, reproductive health and complex humanitarian emergencies), including both field staff and those at head offices. To appropriately contact those with subject matter expertise the following steps were taken

1. Initially, an email was sent to members of the Inter-agency Working Group (IAWG) on Reproductive Health in Crises sub-working group on Research, Data and Heath Information Systems and the general IAWG list serve.

2. In addition, researchers were advised to extend the invitation to others who may have appropriate technical expertise.

3. Lastly, information about the study was presented at the IAWG 13th annual workshop where all attendees who had the appropriate expertise were encouraged to participate and they were followed up with an email invitation.

Every effort was made to invite a mix of people with different backgrounds (clinicians, epidemiologists, public health experts, program leaders and donors) and from different countries (both developed and developing ones) and representing headquarters and field staff so that the mix contains a diversity of views from the wider research community.

### STAGE 3: Scoring of research investment options

Experts were then asked was to score all research questions independently, according to the four agreed criteria. For each of the 35 research questions and each criterion, each expert answered three questions targeted to assess the likelihood of the proposed research to comply with the priority setting criterion (see Additional file 2). This task was completed by all experts. The entire process was conducted and completed via e-mail and web survey between October 14 and December 10, 2011.

Further information on methods related to this part of the priority-setting process are presented elsewhere in greater details.

### STAGE 4: Computations of “research priority scores”

All the experts answered the questions listed in Additional file 2 by “Yes” (1 point) or “No” (0 points). They were also allowed to declare an informed but undecided answer “Yes or No” (0.5 points) or declare themselves insufficiently informed to answer the question “Don’t Know” (missing input).

An overall mean research prioritization score (RPS) was calculated by taking the average of the four criteria. For each research criteria, the average score was computed by taking the average of the subcomponent scores. Questions were ranked in order with the highest RPS. They represent a direct measure of collective optimism of the scorers. Each of the 35 listed research questions received four intermediate scores (each ranging between 0–1.00).

### Assessment of agreement between scorers

As a measure of agreement among scorers, the Average Expert Agreement (AEA) was defined as the average of the highest proportion of the 12 subcomponents among scorers for each of the 28 questions asked. The AEA is informing us, for an average question, what proportion of scorers gave the same most frequent answer.

“CHNRI methodology has the ability to expose the issues of greatest agreement and controversy. This allows more focused discussion among experts following this exercise, and informs the investors and policy makers about the amount of controversy that surrounds each research question. The datasets that CHNRI methodology produces are not appropriate for application of the usual Kappa agreement statistics” [37].

### Advantages and limitations of the CHNRI methodology

The applied CHNRI methodology proved to be helpful to systematically list and score a very large number of specific research questions, as shown recently in exercises on research prioritization for preventing stillbirths, intrapartum-related neonatal deaths, newborn infection, and preterm and low birth rate, zinc deficiency in children, as well as mental health and disability [29,33-45].

Additionally, the CHNRI process is systematic, transparent, that uses an a priori, well-defined context and criteria. This process engages multiple stakeholders and allows for scoring that limits influence of strong-minded individuals on the rest of the scorers and quantitatively determines research priorities and degree of agreement or disagreement between experts.

Although the CHNRI methodology offers many advantages, there are still limitations. Though initial list of questions evaluated by the experts represents a systematic determination of potential priorities, there may be additional questions not included on this list that warrant consideration. Additionally a concern that has been raised previously, is that he result of the CHNRI process could represent a biased opinion of a very limited group of involved experts [33]. The number of people globally who possess enough experience, expertise and knowledge on this neonatal health and complex humanitarian emergencies to be able to judge a very diverse spectrum of research questions is rather limited. As this is a limited pool of people, we feel reassured that a response from 28 participants falls within the range of other exercises [37,38] and represented a diverse group of people.

### Validation of CHNRI methodology

The CHNRI methodology has been used in many previous studies with success. Additional information on the validity of the methods can be found in the web appendices of “Setting Research Priorities to Reduce Almost One Million Deaths from Birth Asphyxia by 2015” [37].

## Abbreviations

AEA: Average expert agreement score; CDC: United States centers for disease control and prevention; CHRNI: Child health nutrition research initiative; CMR: Crude mortality rate; DHS: Demographic health surveys; ECHO: The European Union Humanitarian Aid and Civil Protection; HIV/AIDS: Human Immunodeficiency Virus/Acquired Immunodeficiency Syndrome; IAWG: Inter-Agency Working Group; IDP: Internally Displaced Person(s); MDG: Millennium development goal; MICS: Multiple indicator cluster surveys; NGO: Non-Governmental Organization; RPS: Research prioritization score; U5MR: Under 5 mortality rate; UNFPA: United Nations Population Fund; UNHCR: United Nations high commissioner for refugees; UNICEF: United Nations International Children’s Fund.

## Competing interests

There are no competing financial or non-financial interests or conflicts of interest by any of the authors of the study.

## Authors’ contributions

All authors meet the Uniform Requirements of Manuscripts Submitted to Biomedical journals. RA contributed to the study conception, study design, interpretation of the data, drafted and revised the manuscript and approved the final version. DM and BT contributed to the study design, analysis, and interpretation of the data, drafted and revised the manuscript and approved the final version. KK and JL contributed to the study conception, study design, interpretation of the data, revised the manuscript and approved the final version. CB contributed to the study design, interpretation of the data, revised the manuscript, and approved the final version. SS contributed to the interpretation of the data, drafting and revision of the manuscript and approval of the final version. All authors read and approved the final manuscript.

## Supplementary Material

Additional file 1 **Composition of affiliations of the group of technical experts.** All participation in this particular CHNRI exercise was voluntary and carried out without funding support. All the experts who were invited to participate in that exercise had expertise on neonatal health, child health, reproductive health, and complex humanitarian emergencies. More than one participant may have been from each affiliation. Click here for file

Additional file 2 **Criteria used for ranking questions adapted from Child Health and Nutrition Research Initiative methodology **[29,46]**.** Click here for file
