# Supplementary material for: Neonatal survival in complex humanitarian emergencies: setting an evidence-based research agenda
Source: Confl Health. 2014 May 20;8:8. doi: 10.1186/1752-1505-8-8 (PMC4057580; doi:10.1186/1752-1505-8-8)
Supplement: Additional file 1 — Composition of affiliations of the group of technical experts. All participation in this particular CHNRI exercise was voluntary and carried out without funding support. All the experts who were invited to participate in that exercise had expertise on neonatal health, child health, reproductive health, and complex humanitarian emergencies. More than one participant may have been from each affiliation. [file 1752-1505-8-8-S1.doc]

Appendix 2: Composition of affiliations of the group of technical experts

All participation in this particular CHNRI exercise was voluntary and carried out without funding support. All the experts who were invited to participate in that exercise had expertise on neonatal health, child health, reproductive health, and complex humanitarian emergencies. More than one participant may have been from each affiliation.

|  |  | **Affiliation** | | | |  | | |  |
| --- | --- | --- | --- | --- | --- | --- | --- | --- | --- |
| 1 |  | Aga Khan University, Nairobi | | | |  | | |  |
| 2 |  | ASPRO |  | |  | | |  |  |
| 3 |  | FHI 360 |  | |  | | |  |  |
| 4 |  | Harvard School of Public Health | | | |  | | |  |
| 5 |  | Ipas |  | |  | | |  |  |
| 6 |  | IRC |  | |  | | |  |  |
| 7 |  | Johns Hopkins Bloomberg School of Public Health | | | | | | | |
| 8 |  | JSI Research & Training Institute | | | |  | | |  |
| 9 |  | Medicos sin Fronteras | |  | | |  | |  |
| 10 |  | Save the Children | |  | | |  | |  |
| 11 |  | STC Uganda | |  | | |  | |  |
| 12 |  | The European Union Humanitarian Aid and Civil Protection (ECHO) | | | | | | | |
| 13 |  | The United Nations Children's Fund (UNICEF) | | | | | | |  |
| 14 |  | U.S. Centers for Disease Control and Prevention | | | | | | | |
| 15 |  | United Nations High Commissioner for Refugees (UNHCR), | | | | | | | |
| 16 |  | United Nations Population Fund (UNFPA) | | | | | | |  |
| 17 |  | Women's Refugee Commission | | | |  | | |  |
| 18 |  | World Vision International | | | |  | | |  |
|  |  |  | | | |  | | |  |
