# Supplementary material for: Neonatal survival in complex humanitarian emergencies: setting an evidence-based research agenda
Source: Confl Health. 2014 May 20;8:8. doi: 10.1186/1752-1505-8-8 (PMC4057580; doi:10.1186/1752-1505-8-8)
Supplement: Additional file 2 — Criteria used for ranking questions adapted from Child Health and Nutrition Research Initiative methodology [29,46]. [file 1752-1505-8-8-S2.doc]

**Box 1: Criteria used for ranking questions adapted from Child Health and Nutrition Research Initiative methodology**

**
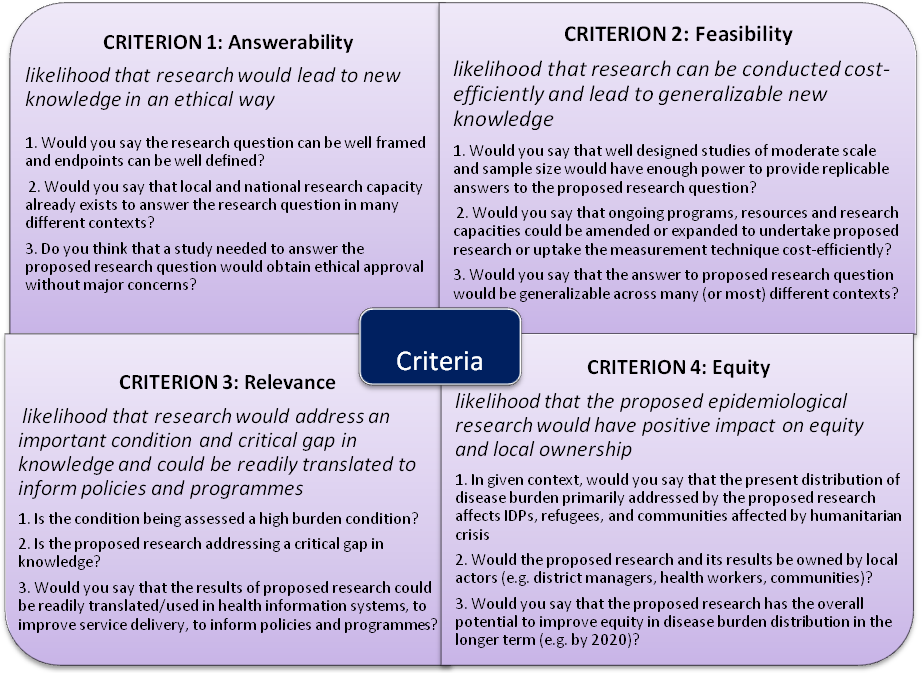
**
